# Special Issue “Wearable and BAN Sensors for Physical Rehabilitation and eHealth Architectures”

**DOI:** 10.3390/s21248509

**Published:** 2021-12-20

**Authors:** Maria de Fátima Domingues, Andrea Sciarrone, Ayman Radwan

**Affiliations:** 1 Instituto de Telecomunicações, Campus Universitário de Santiago, 3810-193 Aveiro, Portugal; aradwan@av.it.pt; 2 Department of Electrical, Electronic, Telecommunications Engineering, and Naval Architecture (DITEN), University of Genoa, Via dell’Opera Pia 13, 16145 Genoa, Italy; andrea.sciarrone@unige.it

## 1. Introduction

The demographic shift of the population toward an increased number of elder citizens, together with the sedentary lifestyle we are adopting, is reflected in the increasingly debilitated physical health of the population. The resulting physical impairments require rehabilitation therapies that may be assisted by the use of wearable sensors or body area network sensors (BANs). The use of novel technology for medical therapies can also contribute to reducing the cost of healthcare systems and decrease the patient overflow in medical centers. Sensors are the primary enablers of any wearable medical device, with a central role in eHealth architectures. The accuracy of the acquired data relies on the sensors; hence, when considering wearable and BAN sensing integration, they must prove to be accurate and reliable solutions.

This Special Issue (SI) focuses on the current state-of-the-art BANs and wearable sensing devices for the physical rehabilitation of impaired or debilitated citizens. Both original research papers and review articles describing the current state-of-the-art were considered for publication. We believe that this SI will provide the reader with an overview of the present status and a future outlook of the aforementioned topics.

The contributions to this SI resulted in a collection of 10 published manuscripts reporting on the advances in research related to different sensing technologies (optical or electronic) and body area network sensors (BANs); their design and implementation; advanced signal processing techniques and the application of these technologies in areas such as physical rehabilitation, robotics, medical diagnostics and therapy.

A short overview of the collection of papers accepted for publication in this SI is presented in Section 2.

The guest editors would like to show their token of appreciation to all the authors that contributed to the success of this SI, by providing a set of original papers with a comprehensive and up-to-date overview of a variety of topics, under the umbrella of “Wearable and BAN Sensors for Physical Rehabilitation and eHealth Architectures”.

Furthermore, the work and support of the academic editors and reviewers is highly appreciated. They were a key factor to guarantee the high quality and the scientific rigor of the published manuscripts and, consequently, of this Special Issue.

## 2. Contributed Papers

The manuscripts accepted for publication in this SI mirror the relevance of the topic for the research community, and the vast field of research that still exists to be explored to enhance the wearable and BAN solutions for physical rehabilitation applications and eHealth architectures.

The authors of [1] presented the design and study of different mobility aids (smart walker) configurations, targeting the population who suffers from visual and mobility impairments. In this study, the authors explored different technologies and software configurations to evaluate the performance of the different solutions and reach the conclusion that there is not one configuration that will be suitable for all. Instead, they found that multiple and different choices of sensors can provide a similar user experience. Nevertheless, emphasis should be given to the fact that active sensors (ultrasonic distance sensors or infrared depth cameras) provide a better accuracy for the localization of objects/obstacles [1]. The authors also reach the conclusion that it is necessary to perform a holistic evaluation of the walker in terms of its end-to-end performance, and that the user interface is of big importance to the overall performance of a smart walker [1].

Bezuidenhout et al. presented a study on the reliability of Actigraph GT3X+ (AG) accelerometers to detect gait parameters. The devices were worn on the hip and on the ankle by thirty healthy individuals walking in a straight line and turning at different speeds. As a reference, a Stepwatch (SW) activity monitor was used, which was attached to the right ankle [2]. The authors found that the AG placed on the ankle provided the best accuracy for the detection of steps at speeds less than 0.6 m/s, and for speeds above this value, the detection of steps was only possible by applying a low frequency extension filter (LFEF). The hip worn AG presented accuracy above 87% for gait speeds <0.1 m/s, which was considerably degraded with an increase in the gait speed. The authors’ findings suggest that the location where the sensor is placed, together with the type of data filters used, are key factors that influence the accuracy of the step counts [2].

In the third contribution to this SI, the authors Di Tocco et al. presented their study on the development of wearable solutions for unobtrusive cardio-respiratory monitoring [3]. The proposed solution is based on four conductive textiles sensors, which are placed on the user’s chest. The deformation induced on the sensors, by the expansion and contraction of the rib cage due to the respiratory cycle, provides reliable information, from which the users breathing activity can be inferred [3]. As for the heart rate, the authors used an IMU placed on the left-hand side of the chest. In the trials performed with the wearable system based on a multi-sensor configuration, the authors found that it provided consistent measures for the respiratory and heart rate for all the subjects and scenarios tested [3].

The authors from [4] presented a study on the long- and short-term effects of a scapular exercise on the function and pain in individuals with rotator-cuff-related pain syndrome (RCS) and anterior shoulder in-stability (ASI) [4]. The results presented were the outcome of a study performed in one hundred and eighty-three patients, from which 171 suffered from RCS and 66 from ASI. The assessment of the shoulder pain and function was performed during the implantation of the structure exercise protocol at its beginning, 4th week and at the 2-year follow up [4]. The authors found a substantial improvement in the 4-week assessment, and not a major difference between the 4th week and the 2nd year follow up, which is a valuable indicator of the positive impact of the exercise protocol implemented in the short and long term [4].

The authors of [5] presented a study on the physiological parameters (with particular focus on the heart rate variability (HRV)) that can be extracted from wearable devices to detect stress levels in car drivers. The authors developed a predictive model based on different machine learning (ML) methodologies such as K-Nearest Neighbor (KNN), Random Forest (RF), among others that is able to classify the stress level extracted from ECG-derived HRV features [5]. The techniques proposed by the authors show that the HRV features can act as markers for stress level detection, achieving a recall of 80% with the ML models proposed [5].

The contribution by Rutkowski et al., a study focusing on the use of physical activity sensors (such as the SenseWear armband) in patients with chronic obstructive pulmonary disease (COPD), was presented to monitor their activity level in day-to-day life and for the duration and intensity of physical activity. The approach implemented by the authors allowed them to understand the daily activity of the patients and if they undertake the prescribed unsupervised physical activity, and additionally, to understand the strengths and weaknesses of the selected type of sensors [6]. Based on the sensors’ feedback, in terms of resting time, number of steps, physical activity level and energy expenditure (kcal), the authors did not find a significant difference (statistically) between the non-supervised and supervised physical activity days. Furthermore, the authors found that the use of this type of sensor may improve the patient’s self-esteem and motivate them to continue physical activity and, in that way, improve their health condition [6].

Another work devoted to the human activity recognition (HAR), using smartwatch inertial sensors, was presented by the authors of [7]. The authors study the performance of three algorithms for the out-of-distribution (OOD) detection of activity classes data that are not present in the training data of the ML [7]. The authors collected a new data set (SPARS9x) from inertial smartwatch sensors worn by 20 volunteers, first performing supervised physical exercises and, after, performing other unrelated physical movements (OOD). From this analysis, the authors showed that traditional algorithms outperform deep learning algorithms for this particular case of OOD detection for HAR [7].

The valuable contribution by Liu et al., for the success of this SI, was also focused on the use of wearable inertial sensors, but for the ambulatory detection of the human gait phase [8]. The analysis of gait parameters, such as its phase, is of extreme importance in the diagnose of diseases (e.g., Alzheimer’s, Parkinson’s) or post-surgery rehabilitation evolution. The authors proposed a methodology to infer the gait phase, based on the angular velocity provided by inertial sensors, associated to a Hidden Markov Model (HMM) used to segment the gait phases. The outcome of the experiments implemented by the authors demonstrate that their model is able to accurately recognize the gait phase segmentation [8].

The authors of [9] presented their study on the use of multiple sensing technology (mostly miniature wearable inertial sensor nodes) allied to the extended Kalman filter (EKF) method, to evaluate the training performance (stroke posture, rhythm) of kayakers. The authors, based on the kinematic information retrieved by the sensors, resort to ML algorithms to distinguish the stroke cycle phases, providing a comprehensive evaluation of the kayaker’s motion on a real scenario, with a stroke phase match of up to 98% (validated by videography) [9]. The techniques, proposed by these authors, can supply the needed quantitative data for coaches and athletes to improve their physical performance [9].

The review paper presented by Vilela et al. discusses the innovative and relevant topic of fog-computing in the area of eHealth [10]. The authors present a review of eHealth applications using fog-computing. The paper focuses on the existing solutions in the literature that use fog-cloud computing with very tight requirements in terms of latency, security and energy efficiency. An architectural overview of communication technologies is elaborated. The paper concentrates on highlighting the gains in the performance of fog networking, in terms of delay and the amount of data. Finally, the authors shed light on challenges in the area for future research efforts [10].

## 3. Outlook and Prospects

The set of papers published in this SI is just a small representation of the current research interest regarding the use of wearable and BAN sensors for physical rehabilitation and activity monitoring. As the field for wearable sensors evolves, improving its range of detection, resolution and accuracy, new applications and higher accuracy detection levels can be achieved by widening the application of these technologies even more. When allied to ML algorithms, other emerging fields of applications can be sought, such as the digital twin features, where there is a vast area of research still to be pursued.
